# Characterization of Metabolites in Plasma, Urine and Feces of Healthy Participants after Taking Brahmi Essence for Twelve Weeks Using LC-ESI-QTOF-MS Metabolomic Approach

**DOI:** 10.3390/molecules26102944

**Published:** 2021-05-15

**Authors:** Genet Minale, Tongchai Saesong, Prapapan Temkitthawon, Neti Waranuch, Nitra Nuengchamnong, Krongkarn Chootip, Natakorn Kamkaew, Teeraporn Kongbangkerd, Jinutda Engsuwan, Kornkanok Ingkaninan

**Affiliations:** 1 Centre of Excellence in Cannabis Research, Department of Pharmaceutical Chemistry and Pharmacognosy, Faculty of Pharmaceutical Sciences and Center of Excellence for Innovation in Chemistry, Naresuan University, Phitsanulok 65000, Thailand; genetminaleye59@nu.ac.th (G.M.); tongchais56@nu.ac.th (T.S.); prapapant@nu.ac.th (P.T.); 2 Cosmetics and Natural Products Research Center, Department of Pharmaceutical Technology, Faculty of Pharmaceutical Sciences and Center of Excellence for Innovation in Chemistry, Naresuan University, Phitsanulok 65000, Thailand; netiw@nu.ac.th; 3 Science Laboratory Centre, Faculty of Science, Naresuan University, Phitsanulok 65000, Thailand; nitran@nu.ac.th; 4 Department of Physiology, Faculty of Medical Sciences, Naresuan University, Phitsanulok 65000, Thailand; krongkarnc@nu.ac.th (K.C.); natakorn.ka@up.ac.th (N.K.); 5 Unit of Excellence in Clinical Research, Division of Physiology, School of Medical Sciences, University of Phayao, Phayao 56000, Thailand; 6 Department of Agro-Industry, Faculty of Agriculture Natural Resources and Environment, Naresuan University, Phitsanulok 65000, Thailand; teerapornk@nu.ac.th; 7 Department of Cosmetic Sciences, School of Pharmaceutical Sciences, University of Phayao, Phayao 56000, Thailand; jinutda.en@up.ac.th

**Keywords:** *Bacopa monnieri*, metabolomics, brahmi essence, LC-ESI-QTOF-MS, OPLS-DA

## Abstract

Brahmi essence, developed from *Bacopa monnieri* (L.) Wettst. standardized extract and mulberry juice, was proven to improve the memory speed of healthy participants aged 55–80 years old, following a 12-week dietary program. However, the metabolites have not yet been reported. Our objective was to characterize the altered metabolites in the plasma, urine, and feces of healthy volunteers after consumption of Brahmi essence for 12 weeks, using the LC-MS metabolomics approach. The altered metabolites were selected from OPLS-DA S-plots; 15 metabolites in the plasma, 7 in the urine, and 17 in the feces samples were tentatively identified by comparison with an online database and literature. The metabolites in the plasma samples were in the classes of amino acids, acylcarnitine, and phospholipids. Benzeneactamide-4-O-sulphate and 3-hydroxyhippuric acid were found in urine samples. The metabolites in the class of amino acids, together with jujubogenin and pseudojujubogenin, were identified in the fecal samples. The aminoacyl-tRNA, aromatic amino acids, and branched-chain amino acid biosynthetic pathways were mainly related to the identified metabolites in all three samples. It could be implied that those metabolites and their pathways might be linked with the effect of Brahmi essence on memory speed.

## 1. Introduction

*Bacopa monnieri* (L.) Wettst. (Bacopa or Brahmi) is an Ayurvedic medicinal plant in the family Plantaginaceae which grows in wet soils and is distributed in Asia, United States, Africa, and Australia [1,2]. In addition to its traditional use for memory and intellectual enhancement, Brahmi has shown neuroprotective effects [3] as an antioxidant [4], an antiepileptic [5], an anti-inflammatory [6], and an anti-cholinesterase [7,8]. It has also been shown to increase cerebral blood flow [9] via vasodilator action [10,11]. Brahmi exhibits those activities with low signs of toxicity, as reported in both animal and human studies [12,13,14].

There are various chemical classes of compounds reported in Brahmi including inter alia saponins, glycosides, phenolic compounds, flavonoids, and steroids. The major active compounds are the dammarane steroidal saponin glycosides: jujubogenin glycosides (bacoside A_3_ and bacopaside X) and pseudojujubogenin glycosides (bacopaside I, bacopaside II and bacopasaponin C) [15,16]. These compounds have been used as markers in the preparation of standardized Brahmi extract [17]. The phytosterols (stigmasterol, β-sitosterol) and flavonoids (apigenin and luteolin) were also reported from Bacopa [15,18]. Bacoside A—which is a mixture of bacoside A_3_, bacopaside II, bacopasaponin C, and bacopaside X—has been reported for nootropic and neuroprotective activities [19,20].

Brahmi extract is used as an ingredient in some dietary supplements. In Thailand, a tablet containing 300 mg of standardized Brahmi extract (BME) was marketed by the Government Pharmaceutical Organization (GPO) of Thailand with a recommended dose of one tablet per day [14]. As an alternative, Brahmi essence has also been prepared from BME and mulberry juice. A randomized, double-blind, placebo-controlled study on healthy participants showed that Brahmi essence improved the memory speed and blood flow [21].

However, studies on the metabolites associated with these effects have not yet been reported. This was our objective: to identify the metabolites in the plasma, urine, and feces of healthy participants after 12 weeks of consumption of Brahmi essence using an LC-MS--based metabolomics approach.

Metabolomics is a platform used to analyze metabolites in biological samples by combining instrumental techniques and bioinformatics [22,23]. It can be applied to monitor the change of metabolites in complex biological matrices in response to interventions or disturbances of either chemicals or disease processes [24]. In our study, we applied this platform to characterize metabolites in human plasma, urine, and feces.

## 2. Results and Discussion 

The Brahmi essence and placebo were analyzed using LC-ESI-QTOF-MS before analysis of plasma, urine, and feces samples. As shown in Figure S1a,b, the five saponins glycosides (bacoside A_3_, bacopaside II, bacopaside X, bacopaside I, and bacopasaponin C) were present in the Brahmi essence, but not in the placebo. These compounds have been reported to be responsible for the pharmacological effect of Brahmi [19,20]. As mulberry is the ingredient of both placebo and Brahmi essence, some chemical constituents of mulberry—such as cyanidin 3-O-rutinoside, cyanidin 3-O-glucoside and rutin—were found in both Brahmi essences and placebo (Figure S1a and b and Table S1, Supplementary Material).

The metabolites in the plasma, urine, and feces samples were characterized in this study. Before any treatment, the participants (*n* = 48) were asked to give fasted blood, urine, and feces samples. Their memory was also assessed using computerized tests. These data were used as our baseline. After that, all participants consumed placebo for two weeks to provide the placebo run-in data. Biological samples and memory assessment data were collected at the end of this period. The advantages of the placebo run-in were that it allowed us to observe the compliance of the volunteers for the duration of the study, study the safety of placebo, monitor for adverse events, and provide the second baseline of blood biochemistry values. After the placebo run-in, the participants were randomly sorted into two groups: treatment group (*n* = 24) and placebo group (*n* = 24). However, three volunteers were discontinued from the treatment group, leaving 21 participants. The volunteers in the treatment group (*n* = 21) consumed Brahmi essence and those in the placebo group (*n* = 24) took placebo for 12 weeks. The 12-week period of consumption was designed based on the previous report by Peth-Nui et al. [14] stating that the standardized Brahmi extract led to improvements in the quality and memory speed of healthy volunteers after 12 weeks of consumption. Blood, urine, and feces samples—together with data from memory assessments—were collected at the end of 12 weeks (Figure S2, Supplementary Material). All the biological samples were analyzed using LC-ESI-QTOF-MS. The positive ionization mode was used, as it gave more peaks than the negative ionization mode. The data from the placebo run-in and the data after 12 weeks of treatment with either Brahmi extract or placebo were used to characterize the metabolites. Preliminary analysis of some plasma and urine samples by both negative and positive MS modes indicated similar patterns of metabolite profiles with higher intensities of *m/z* in positive mode. In addition, some peaks were only detectable in positive MS modes and could not be observed in negative mode. Based on these results and the fact that it was more practical to use one set of MS data for OPLS-DA, the positive ionization mode was selected to analyze all samples. However, one limitation of our study is that there could have been additional data on metabolites that we did not observe—especially those only observable in negative ionization mode in LC-MS analysis. 

### 2.1. Analysis of Metabolites in Plasma Samples from Healthy Participants after Taking Brahmi Essence for 12 Weeks

**Figure 1:**
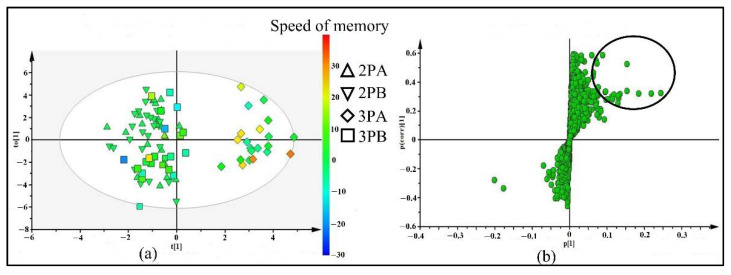
OPLS-DA score plot (**a**) and S-plot (**b**) of 4 groups of plasma samples including: 2PA (plasma samples of 21 participants who took placebo for 2 weeks), 2PB (plasma samples of 24 participants who took placebo for 2 weeks), 3PA (plasma samples of 2PA who took Brahmi essence for 12 weeks) and 3PB (plasma samples of 2PB who continued taking placebo for 12 weeks). The color scale in score plot shows the degree of memory speed, where red represents the highest change of memory speed and blue represents the lowest change of memory speed.

Four groups of plasma samples were identified with codes: 2PA for samples from 21 participants who took the placebo for two weeks, 3PA for samples from 2PA participants who then took the Brahmi essence for the subsequent 12 weeks, 2PB for the samples from 24 participants who took the placebo for two weeks, and 3PB for the samples from 2PB participants who continued to take the placebo for the subsequent 12 weeks. LC-ESI-QTOF-MS and XCMS analysis data were used for supervised multivariate analysis (OPLS-DA). As shown in Figure 1a, the OPLS-DA score plot separated the 3PA group from 2PA, 2PB, and 3PB groups with R^2^(X) = 0.36, R^2^(Y) = 0.87, and Q^2^ = −0.16. This showed that there were changed or altered plasma metabolites after taking Brahmi essence for 12 weeks. In addition, as shown in the score plot, most of the participants who consumed Brahmi essence (3PA group) showed improvements in memory speed compared to participants who took placebo (3PB). Those variables (metabolites) that contributed to the group separation were selected from the OPLS-DA S-plot (Figure 1b). The features with p[1] (≥0.5) and p(corr)[1] (≥0.2) cutoff values were marked and filtered as altered metabolites for further identification. In addition, those filtered metabolites were likely associated with the effect of the Brahmi essence on improving the memory speed. 

As shown in Table 1, 18 features (1–18) were selected from the S-plot for further identification, and 15 of them were tentatively identified by comparing their molecular weight and fragmentation pattern with the databases. More spectroscopic data will be needed for the unidentified (UI) metabolites. 

Branched chain amino acids (valine (*m/z* 114.0863, Rt = 3.1 min) (**2**), leucine/isoleucine (*m/z* 132.1019, Rt = 3.9 min) (**5**)) and aromatic amino acids (tyrosine (*m/z* 182.0810, Rt = 3.5 min) (**4**), phenylalanine (*m/z* 166.0861, Rt=5.3 min) (**6**), and tryptophan (*m/z* 205.0972, Rt = 6.7 min) (**7**)) were tentatively identified as differential metabolites.

The acylcarnitines (acetylcarnitine (*m/z* 204.1241, Rt = 3.2 min) (**3**), octanoylcarnitine (*m/z* 288.2169, Rt = 13.1 min) (**8**), decanoylcarnitine (*m/z* 316.2480, Rt = 15.7 min) (**9**)), palmitoylcarnitines (*m/z* 400.3415, Rt = 22.7 min) (**13**), and oleoylcarnitines (*m/z* 426.3572, Rt = 23.3 min) (**14**), which showed similar fragment ions at *m/z* 85 (-C_4_H_5_O_2_^+^) and *m/z* 144 (-C_7_H_14_NO_2_^+^), were tentatively identified as metabolites in plasma samples. Acylcarnitines are involved in neuroprotection—stabilizing the membrane composition through the modulation of lipid synthesis, enhancing the mitochondrial function, ameliorating antioxidant activity, and modulating cholinergic neurotransmission [25]. They also have the potential to regulate brain energy metabolites and reverse the metabolic change of aging [26].

Lysophosphatidylcholines (LysoPC) (LysoPC (16:1 (9Z)/0:0) (*m/z* 494.3238, Rt = 22.6 min) (**12**), lysoPC (20:4 (8Z, 11Z, 14Z, 17Z)/0:0) (*m/z* 544.3398, Rt = 23.7 min) (**15**), lysoPC (18:2 (9Z, 12Z)/0:0) (*m/z* 520.3392, Rt = 23.8 min) (**16**), lysoPC (16:0/0:0) (*m/z* 496.3398, Rt = 25.5 min) (**17**) and lysoPC (18:1) (*m/z* 522.3556, Rt = 26.6 min)(**18**)) were the other tentatively identified metabolites. LysoPCs are characterized by a unique fragmentation pattern at *m/z* 104 (-C_4_H_8_O_3_) and *m/z* 184 (-C_5_H_15_NO_4_P^+^). Plasma lysoPCs are used as a transporter of Docosahexaenoic acid (DHA) to the brain [27]. DHA reduces the production of Aβ in the brain and prevents the development of Alzheimer’s disease [28].

**Figure 2:**
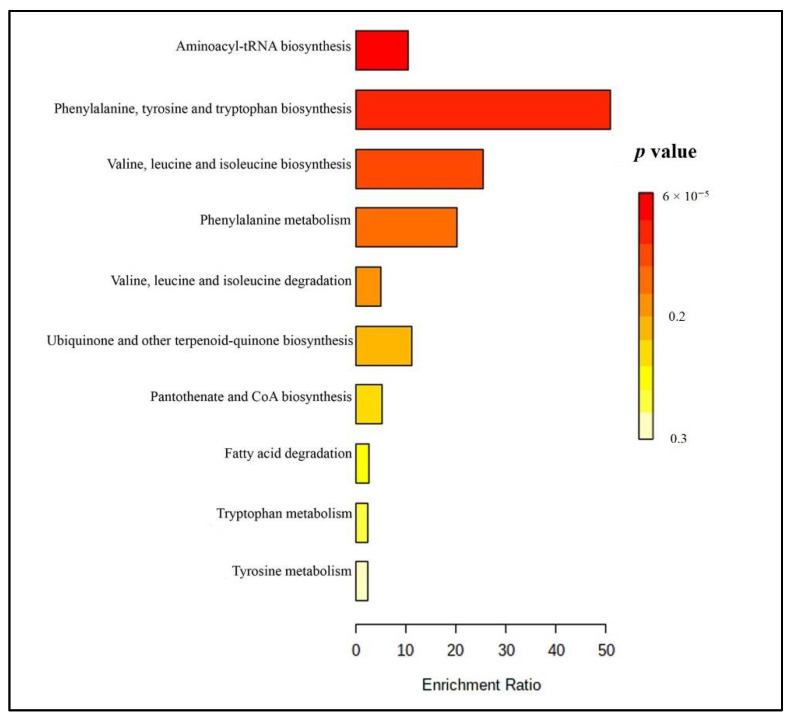
Summary plot of overrepresentation analysis (ORA) for plasma samples (the color represents: red-lower *p*-values and yellow-higher *p* value).

The altered metabolic pathways were analyzed using Metaboanalyst 4.0. The results showed 10 metabolic pathways related to the tentatively identified metabolites in the plasma samples (Figure 2 and Table S3a, Supplementary Material). The major altered pathways were (1) aminoacyl-tRNA biosynthesis, (2) phenylalanine, tyrosine, and tryptophan biosynthesis, (3) valine, leucine, and isoleucine biosynthesis and degradation, and (4) phenylalanine metabolism. These pathways play a role in the production of neurotransmitters, protein synthesis, and energy production [29,30,31]. Those metabolites and pathways might be linked to the effect of Brahmi essence on improving memory speed and blood flow.

### 2.2. Analysis of Metabolites in Urine Samples from Healthy Participants after Taking Brahmi Essence for 12 Weeks

**Figure 3:**
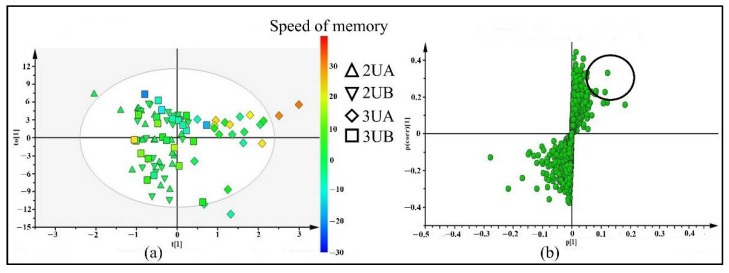
OPLS-DA score plot (**a**) and S-plot (**b**) of 4 groups of urine samples including 2UA (urine samples of 21 participants who took placebo for 2 weeks), 2UB (urine samples of 24 participants who took placebo for 2 weeks), 3UA (urine samples of 2UA who took Brahmi essence for 12 weeks) and 3UB (urine samples of 2UB who continued taking placebo for 12 weeks). The color scale in score plot shows the degree of memory speed, where red color represents the highest change in memory speed and blue color represents the lowest change in memory speed.

The four groups of urine samples from LC-ESI-QTOF-MS and XCMS analysis—2UA (urine samples from 21 participants who took placebo for 2 weeks), 3UA (urine samples of 2UA participants who took Brahmi essence for 12 weeks), 2UB (urine samples of 24 participants who took placebo for 2 weeks), and 3UB (urine samples of 2UB participants who continued taking placebo for 12 weeks)—were used for OPLS-DA analysis. As shown in the OPLS-DA score plot (Figure 3a), the 3UA group were separated from 2UA, 2UB, and 3UB with scores for R^2^(X) = 0.42, R^2^(Y) = 0.7, and Q^2^ = −0.51, which implies that there were metabolites in urine samples that altered after chronic consumption of Brahmi essence. Those participants of 3UA group also showed improvements in memory speed as compared to the placebo group (Table S2, Supplementary Material). The metabolites from the OPLS-DA S-plot (Figure 3b) with p[1] (≥0.5) and p(corr)[1] (≥0.2) cutoff values were filtered and selected as discriminating variables for further identification. The Brahmi essence-treated group showed improvement in memory speed when compared with the placebo group (Table S2). Thus, those selected metabolites were likely associated with the enhancement of memory speed. As shown in Table 1, 10 features (**19**–**28**) were selected from the S-plot for further identification and 7 of them were tentatively identified by comparing their molecular weight and fragmentation pattern with the literature and databases. For the identification of unidentified (UI) metabolites, further information would need to be collected through other spectroscopic techniques. 

One of the metabolites tentatively identified as a differential metabolite was creatinine (*m/z* =114.0675, Rt = 3.1 min) (**19**), which is a common metabolite in urine. Even though creatinine slightly increased in the Brahmi essence group, the plasma creatinine levels of all volunteers were still in the normal range, indicating safety of both placebo and Brahmi essence [21].The metabolite proline betaine (stachydrine) (*m/z* = 144.1027, Rt = 3.4 min) (**20**), which is a urinary biomarker for citrus fruit consumption, was detected as a differential metabolite in urine samples [32]. Phenylalanine (*m/z* =166.0870, Rt = 7.4 min) (**21**), and the tripeptide valine-leucine-Serine (*m/z* = 318.2025, Rt = 8.8 min) (**22**) were also tentatively identified as metabolites that were altered by the consumption of Brahmi essence. Heptonylcarnitine at *m/z* =156.1915 and Rt =9.1 min (**23**), which are linked to the beta oxidation of fatty acids and energy metabolism, were detected as differential metabolites. The other urinary metabolites at *m/z* =232.0283 and Rt =9.5 min (**24**) and at *m/z* = 196.0611 and Rt = 13.58 min (**25**) were tentatively identified as benzeneacetamide-4-O-sulphate and 3-hydroxy hippuric acid, respectively. The latter metabolite is a microbial metabolite derived from polyphenols and flavonoids in human urine [33]. 

**Figure 4:**
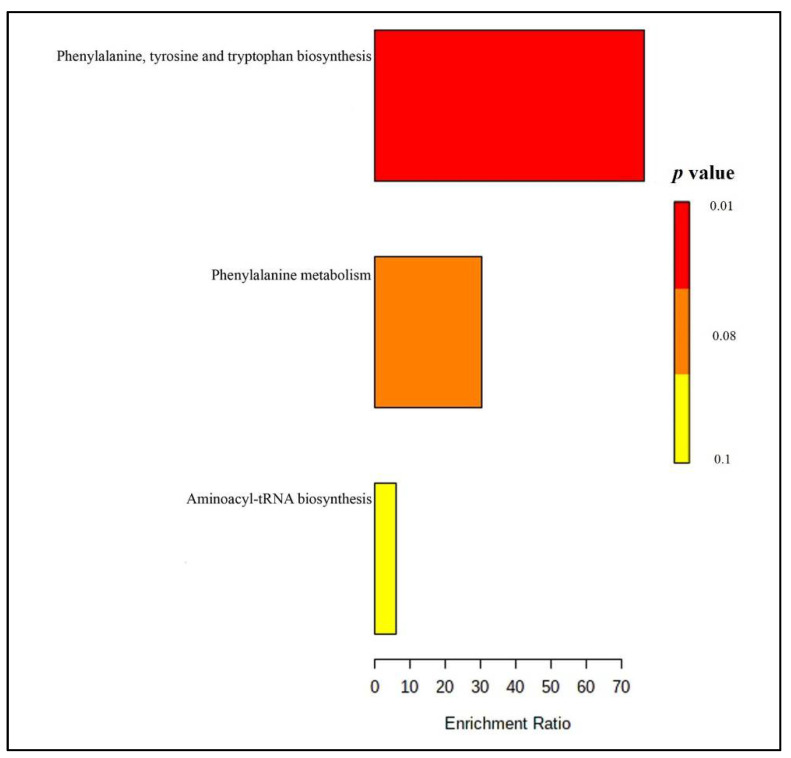
Summary plot of overrepresentation analysis (ORA) for urine samples (the color represents red-lower *p*-values and yellow-higher *p* value).

The tentatively identified metabolites in the urine samples were subjected to metabolic pathway analysis by Metaboanalyst 4.0. The results showed that phenylalanine, tyrosine, and tryptophan biosynthesis (as well as phenylalanine metabolism) (Figure 4 and Table S3b) were the main pathways altered after taking the Brahmi essence for 12 weeks.

### 2.3. Analysis of Metabolites in Feces Samples from Healthy Participants after Taking Brahmi Essence for 12 Weeks

**Figure 5:**
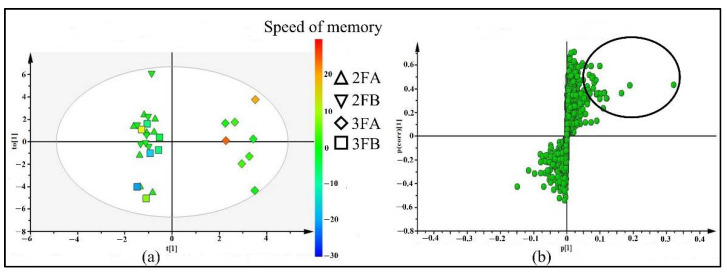
OPLS-DA score plot (**a**) and S-plot (**b**) of 4 groups of fecal samples, including 2FA (fecal samples of 8 participants who took placebo for 2 weeks), 2FB (fecal samples of 7 participants who took placebo for 2 weeks), 3FA (fecal samples of 2FA who took Brahmi essence for 12 weeks) and 3FB (fecal samples of 2FB who continued taking placebo for 12 weeks). The color scale in score plot shows the degree of memory speed where red color represents the highest change of memory speed and blue color represents the lowest change of memory speed.

The fecal samples of participants from four groups obtained from LC-ESI-QTOF-MS and XCMS analysis—coded as 2FA (fecal samples of 8 participants who took placebo for 2 weeks), 3FA (fecal samples of 2FA group participants who took Brahmi essence for 12 weeks), 2FB (fecal samples of 7 participants who took placebo for two weeks), and 3FB (fecal samples of 2FB group participants who continue to took placebo for 12 weeks)—were imported to SIMICA for OPLS-DA analysis. The difficulty of collecting fecal samples from all volunteers at each visit meant that the number of fecal samples was lower than the number of plasma and urine samples. As shown in the OPLS-DA score plot (Figure 5a), the participants in 3FA group were separated from 2FA, 2FB, and 3FB groups with R² (X) = 0.27, R²(Y) = 0.96, and Q^2^ = −0.07. In addition, the 3FA group participants showed improvement in memory speed when compared to the placebo group (Table S2). The discriminating metabolites were selected from OPLS-DA S-plot based on p[1] ≥ 0.5 and p(corr)[1] ≥ 0.2 values (Figure 5b) for further identification. Those metabolites were likely associated with the effect of Brahmi essence on improving memory speed.

A total of 21 metabolites (**29**–**49**) were selected from the S-plot and 17 of them were tentatively identified by comparing their molecular weight and MS/MS fragmentation pattern with databases and literature (Table 1). Other spectroscopic data would be needed to identify the unidentified metabolites (UI).

Lysine (*m/z* 147.1143, Rt = 2.8 min) (**29**), methionine (*m/z* 150.0596, Rt = 4.1 min) (**33**), tyrosine (*m/z* 182.0833, Rt = 4.2 min) (**34**), leucine/isoleucine (*m/z* 132.1002, Rt = 4.7 min) (**39**), phenylaniline (*m/z* 166.0880, Rt = 8.0 min) (**40**), tryptophan (*m/z* 205.0988, Rt = 9.2 min) (**42**), cycloleucine (*m/z* 130.0875, Rt = 3.7 min) (**30**), 2-aminooctanoic acid (*m/z* 160.1347, Rt = 3.7 min) (**31**), 5-aminopentanoic acid (*m/z* 118.0878, Rt = 4.0 min) (**32**), racemethionine (*m/z* 150.0596, Rt = 5.6 min) (**37**) and leucyl proline (*m/z* 229.1561, Rt = 8.5 min) (**41**) were tentatively identified as the metabolites that contributed to the separation of 3FA group from the 2FA, 2FB and 3FB groups.

In addition, features at *m/z* 455.3547, Rt = 20.4 min (**46**), and *m/z* 455.3542, Rt = 27.7 min (**48**) were obtained from the S-plot as differential metabolites. These ions showed similar fragmentation patterns at *m/z* 437, *m/z* 369, *m/z* 315, which might imply that they are isomers. By comparing their fragmentation patterns with the literature [15], compounds **48** and **50** were tentatively identified as jujubogenin and its isomer. Similarly, the ion at *m/z* 473.3656, Rt = 27.7 min (**49**), which showed fragmentation at *m/z* 455, *m/z* 437, *m/z* 369, *m/z* 315, was tentatively identified as pseudojujubogenin. Jujubogenin and pseudojujubogenin are aglycones of the saponin glycosides—which are well-known bioactive constituents of Brahmi. These metabolites might be formed by hydrolysis of jujubogenin and pseudojujubogenin glycosides. Sookying et al. [34] reported the presence of Bacopaside I in rat feces after oral administration of Brahmi extract. Gut microbiota might play an important role in the metabolism of saponin glycosides of Brahmi. 

**Figure 6:**
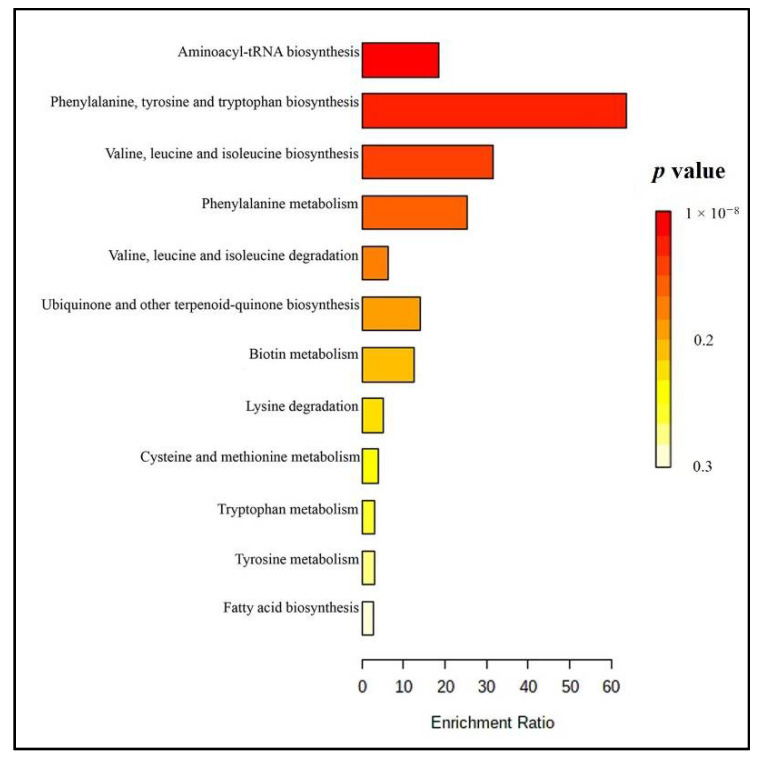
Summary plot of overrepresentation analysis (ORA) for feces samples (red=lower *p*-value, yellow=higher *p* value).

According to our Metaboanalyst analysis (Figure 6 and Table S3c), twelve metabolic pathways were related to the tentatively identified metabolites. Aminoacyl-tRNA biosynthesis, phenylalanine, tyrosine and tryptophan biosynthesis, valine, leucine, isoleucine biosynthesis, and phenylalanine metabolisms were the primary altered pathways observed in samples from those who consumed Brahmi essence for 12 weeks.

In general, the metabolites that we found in plasma, urine and feces samples were associated with memory speed; however, even though it was likely associated with Brahmi essence, the effect could be either direct or indirect. The active compounds or metabolites of the Brahmi essence were not detected in plasma or urine samples, but some were detected in the feces samples.

This may have been due to poor absorption of the active compounds into systemic circulation or the active compounds remaining below the detectable limit of metabolites in the plasma and urine [35]. The large time gap between the intake of the Brahmi essence and the collection of samples may also have been a factor in our being unable to detect the metabolites or active compounds. One limitation of our study was the presence of great variation in the biological samples, stemming from individual differences among the subjects or technical variations which affected the Q^2^ value. The PCA score plot of plasma and urine (Figure S3A,B) showed that some QCs did not cluster each other which might have contributed to such variation. To decrease false positive prediction, we checked the abundance of each selected metabolite in the Brahmi essence-treated group.

## 3. Materials and Methods

### 3.1. Chemicals and Reagents 

Acetonitrile and methanol (LC-MS reagents) were purchased from RCI Labscan Thailand. Ultrapure water was obtained from Millipore MilliQ® Integral 3 Water purification system (Millipore, Bedford, MA, USA). Formic acid (analytical grade) was obtained from Merck (Darmstadt, Germany). Digitoxin and prednisolone were purchased from Sigma-Aldric (St. Louis, MO, USA).

### 3.2. Brahmi Essence and Mulberry Preparation 

Mulberry fruit was obtained from The Queen Sirikit Department of Sericulture, Thailand. Brahmi, which was authenticated by Professor Wongsatit Chuakul, Mahidol University, Thailand, and the specimen (Phrompittayarat 001), which was kept at the herbarium of Mahidol University Thailand, were provided by GPO (Bangkok, Thailand). BME was prepared and standardized as described previously by Kamkaew et al. [11] and Phrompittayarat et al. [35]. Of this extract, 16.03 ± 0.07% (*w/w*) was total saponins, comprising bacoside A_3_ (2.22 ± 0.01%), bacopaside I (3.54 ± 0.01%), bacopaside II (4.68 ± 0.02%), bacopaside X (3.25 ± 0.01%) and bacopasaponin C (2.34 ± 0.01%). The Brahmi essence was prepared from BME (equivalent to 15 mg of total saponins) and mulberry juice, comprising in total 40 mL per dose. The placebo was mulberry juice, also 40 mL per dose. The color, odor and taste of the Brahmi essence were identical with that of the placebo. All the products were pasteurized and kept refrigerated (4 °C) until used. The LC-ESI-QTOF-MS chromatograms and the chemical constituents in the Brahmi essence and the placebo are shown in Figure S1 and Table S1. 

### 3.3. Human Ethical Statement 

The study protocol followed the Declaration of Helsinki guidelines of 1975 and was approved by the Naresuan University Ethical Committee for Human Research (NU-IRB) (protocol code 0898/60) with the research protocol approval certificate number 197/2018.

### 3.4. Design of the Study 

The participants recruited for the study were healthy volunteers between the ages of 55 and 80 who were free from disease (e.g., schizophrenia, dementia, depression, liver disease, kidney disease, diabetes, cancer, stroke, hypertension, and hyperlipidemia) and who did not take any drugs or herbal supplements that might have had an effect on the study. The participants were also selected based on their Thai ethnicity and ability to understand the spoken and written Thai language. An educational level equivalent to the 5^th^ year of primary school was chosen as a minimum, and all participants were able to comprehend and sign the consent form. All participants were informed about the study and gave written informed consent.

The study was designed as a double-blind, placebo-controlled clinical trial (Figure S2). The participants were asked to fast overnight before they came to the trial. At their 1st visit, blood, urine, and feces samples were collected from each volunteer, together with data from memory assessment using a computerized test. All participants were then instructed to take one bottle of placebo per day, 30 min after their evening meal, for two weeks. We called this the placebo run-in period (2nd visit). At the end of this two-week period, blood, urine, and feces samples, along with another memory assessment test, were collected. The volunteers were then randomly divided into the treatment group and the placebo group. The participants in treatment group were requested to drink one bottle of Brahmi essence per day, 30 min after their evening meal, for 12 weeks. The participants in the placebo group were similarly requested to drink one bottle of placebo per day for 12 weeks. At the end of this 12-week period, blood, urine, and feces samples were collected, along with memory assessment test data from each participant (3rd visit).

### 3.5. Plasma, Urine, and Feces Sample Collection

In each visit, the blood samples were collected from each participant in an EDTA tube by medical technicians. The plasma was separated immediately by centrifugation at 1700 rpm at 4 °C for 13 min, and the supernatants then stored at −80 °C until required for analysis. Urine and fecal samples were collected and stored immediately at −80 °C until analysis.

### 3.6. Assessment of Memory Speed

Computerized tests for memory speed were undertaken by each participant. Memory speed was a summation of the time taken to complete word recognition, picture recognition, spatial working memory, and numeric working memory tests. The percent change of response time from the placebo-run-in (2nd visit) to after 12 weeks (3rd visit) was calculated to measure the incremental increase in memory speed of the participants in both groups over that 12-week period [21].

### 3.7. Sample Preparations

#### 3.7.1. Preparation of Brahmi Essence and Placebo for LC-MS Analysis

1 mL of Brahmi essence or placebo was mixed with 2 mL of methanol in 0.1% formic acid. The samples were sonicated for 15 min and centrifuged at 4000 rpm for 5 min. The supernatants were filtered through a 0.2 µm syringe filter and transferred to a vial for LC-MS injection.

#### 3.7.2. Preparation of Plasma, Urine, and Feces Samples for LC-MS Analysis

Frozen plasma samples were thawed on ice and vortexed for 1 min. A 200 µL sample of the plasma was then mixed with 20 µg/mL of prednisolone and 50 µg/mL of digitoxin (internal standards) in cold methanol with 0.1% formic acid in the ratio of 1:3 (sample: solvent) to precipitate the protein. The samples were then kept at 4 °C for 1 h and then centrifuged at 14,000 rpm at 4 °C for 20 min. The supernatant was dried by SpeedVac and reconstituted by methanol in a 10:1 ratio and injected into LC-MS. 

Urine samples were thawed on ice, vortexed for 2 min and centrifuged at 14,000 rpm for 15 min at 4 °C to get a clear solution. Protein precipitations were done by adding cold methanol with 0.1% formic acid with an internal standard solution (20 µg/mL of prednisolone and 50 µg/mL of digitoxin) on 200 µL of urine sample in a 1:2 ratio. The sample was vortexed for 1 min and centrifuged at 14,000 rpm at 4 °C for 20 min. The supernatants were transferred to the HPLC vial and stored at −20 °C until analysis. 

Feces samples were dried by SpeedVac at 40 °C. A sample (10 mg) and internal standard compounds (20 µg/mL of prednisolone and 50 µg/mL of digitoxin) were dissolved by 1 mL of 80% cold methanol mixed with 0.1% formic acid. The solutions were vortexed for 2 min, sonicated for 10 min, and centrifuged at 14,000 rpm for 20 min. The supernatant was transferred to the HPLC vial and stored at −20 °C until injection. 

To check instrumental variability, the pooled samples of plasma, urine and feces samples were analyzed. The pooled samples were obtained by taking 10 µL sample solutions of all participants prepared by the above-mentioned method and mixing them well. The blank samples (methanol) were prepared and injected with the same condition as the samples (Figure S4) to assure that there were no impurities from the LC-MS system. 

### 3.8. LC-ESI-QTOF-MS Analysis Conditions

The chromatographic analysis of all samples was performed using an Agilent 1260 Infinity LC-QTOF-MS dual ESI 6540B model (Agilent Technologies, Singapore) and an Agilent 1260 Infinity Series HPLC system (Agilent, Waldbronn, Germany). A Zorbax Eclipse plus C18 column 4.6 × 100 mm, 3.5 μm with 5 μm guard cartridges (Agilent Technologies, Santa Clara, CA USA) was used for the separation of the plasma samples and a Luna 5u C18 (2) 100 A 150 × 4.6 mm column was used for the urine and feces samples. The mobile phase was composed of 0.1% formic acid in water (*v/v*) (A) and 0.1% formic acid in acetonitrile (*v*/*v*) (B). The linear gradient elution of the mobile phase was started at 5% of solvent B and ended with 95% of solvent B. The run time was 40 min with a postrun of 5 min. The flow rate was set as 0.5 mL/min and column temperature set as 35 °C with injection volume 10 μL. 

The mass spectrometry analysis was performed by an Agilent dual nebulizer with an electrospray ionization (ESI) source set in positive ionization mode with the following detection parameters: drying gas (N_2_) flow rate 10 L/min, drying gas temperature 350 °C, nebulizer pressure 30 psig, capillary 3500 V, skimmer 65 V, octapole RFV 750 V, and fragmentor voltage 100 V. The mass range was set at *m/z* 100–1000. The collision energies of MS/MS were set at 10, 20 and 40 V.

### 3.9. Data Analysis

The data obtained from the LC-ESI-QTOF-MS analysis (. d file) of the plasma, urine, and feces samples of volunteers after placebo run-in and after taking of Brahmi essence or placebo for 12 weeks were converted to mzXML file format using the ProteoWizard software program (http://proteowizard.sourseforge.net). The converted data were analyzed using the XCMS online platform (https://xcmsonline.scripps.edu). The parameters were as follows: feature detection (method = “centwave”, peakwidth = 6, 30 ppm, prefilter = 3500); alignment (mzwid = 0.025, bw = 5). The three data sets generated from the XCMS analysis consisted of peak code, retention time:mass to charge ratio pair (Rt-*m/z*) and ion peak intensity. The data were then normalized by internal standard and exported to SIMCA-P software (version 13.0, Umetrics, Umea, Sweden) for supervised Orthogonal Partial Least Squares Discriminant Analysis (OPLS-DA). All variables were pareto scaled before analysis. In the OPLS-DA S-plot, the X-axis was the contribution/covariance (p[1]), and the Y-axis was reliability/correlation (p(corr)[1]). The features with high p[1] and p(corr)[1] cut off values on the S-plot were marked as the variables that contributed to group separation; these were then selected for further identification. The tentative identifications were done by comparing their molecular mass and fragmentation patterns (MS/MS data) with literature and on-line databases such as the Human metabolomic database (HMDB, http://www.hmdb.ca), METLIN (http://metlin.scripps.edu), ChemSpider (https://www.chemspider.com), LIPID MAPS (https://www.lipidmaps.org), MetFrag (http://metFrag), and Massbank (http://www.massbank.jp). MetaboAnalyst 4.0 software (http://www.metaboanalyst.ca) was used to find the metabolic pathways that were altered by the consumption of the Brahmi essence for 12 weeks.

## 4. Conclusions

The LC-MS metabolomics approach was applied to characterize the altered metabolites in plasma, urine, and feces samples after consumption of Brahmi essence by healthy human participants over a 12-week period. The tentatively identified metabolites might be associated with the improvement of memory speed of the participants, as an effect of having consumed the Brahmi essence. Additionally, the untargeted LC-MS metabolic approach could be used to detect metabolic changes in biological samples that might be directly or indirectly related to the consumption of dietary supplements or other herbal products.

The active compounds and their metabolites in Brahmi essence were not detected in the plasma and urine samples but were detected in the feces sample. The level of active compounds in the plasma and urine samples may have been below the detection limit, or the poor absorption of saponin glycosides may have been a contributing factor to why we did not detect them in our analysis.

## Figures and Tables

**Table 1:** Tentative identification of metabolites in plasma, urine, and feces samples in Brahmi essence-treated groups.

Metabolite ID	Rt (min)	p[1], p(corr)[1] Value	Detected *m/z*	Assigned Ion	Detected MS/MS (% Abundance)	Error (ppm)	Tentative Identification *
**I.**	**Metabolites in plasma**						
1	3.0	0.06, 0.20	101.0030	-	56.9650 (100)	-	UI
2	3.1	0.07, 0.30	118.0863	[M + H] ^+^	72.0807 (100),55.0543 (79),61.0399 (19),102.0112 (1)	−0.4	Valine ^A, E, F^
3	3.2	0.06, 0.30	204.1241	[M + H] ^+^	85.0289 (100), 60.0808 (12), 57.0334 (4), 144.1024 (2)	−6.7	Acetylcarnitine ^A, E, F^
4	3.5	0.06, 0.30	182.0810	[M + H] ^+^	91.0546 (100), 123.0444 (60), 77.0386 (13), 51.0231 (7), 65.0388 (3), 136.0336 (1)	0.9	Tyrosine ^A, E, F^
5	3.9	0.22, 0.30	132.1019	[M + H] ^+^	86.0966 (100)	0.8	Leucine/isoleucine ^A, E, F^
6	5.3	0.15, 0.30	166.0861	[M + H] ^+^	120.0814 (100), 103.0547 (43), 77.0388 (16), 131.0495 (1)	1.5	Phenylalanine ^A, E, F^
7	6.7	0.13, 0.30	205.0972	[M + H] ^+^	146.061 (100), 118.0659 (87), 188.0718 (54), 84.9601 (24), 132.0812 (20), 91.0547 (13), 159.0924 (12), 65.0392 (1)	0.3	Tryptophan ^A, E, F^
8	13.1	0.06, 0.50	288.2169	[M + H] ^+^	85.0293 (100), 60.0815 (9), 229.1462 (6), 57.0707 (4), 144.1014 (2)	0.1	Octanoylcarnitine ^A, E, F^
9	15.7	0.06, 0.50	316.2480	[M + H] ^+^	85.0293 (100), 60.0813 (10), 257.1778 (7), 144.1028 (2), 57.0338 (2), 95.0855 (2), 214.9232 (1)	0.7	Decanoylcarnitine ^A, E, F^
10	16.5	0.12, 0.30	332.0700	-	95.0614 (100), 121.0409 (46), 164.0834 (22), 96.0645 (12)	-	UI
11	18.8	0.08, 0.40	219.1872	-	78.0236 (13.5), 163.1257 (7), 191.1575 (1)	-	UI
12	22.6	0.07, 0.30	494.3238	[M + H] ^+^	184.0749 (44), 104.1075 (42), 311.261 (1), 258.1111 (1)	0.8	LysoPC (16:1(9Z)/0:0) ^A, D^
13	22.7	0.05, 0.40	400.3415	[M + H] ^+^	85.0292 (93), 341.272 (11), 144.1042 (4), 239.2392 (3)	1.6	Palmitoylcarnitine ^A, E, F^
14	23.3	0.05, 0.30	426.3572	[M + H] ^+^	85.0295 (50), 60.0809 (6), 367.2878 (4), 144.1027 (1), 297.2103 (1), 129.0791 (1), 95.0495 (1)	1.4	O-oleoylcarnitine ^A, D, E^
15	23.7	0.07, 0.30	544.3398	[M + H] ^+^	184.0748 (31), 104.1079 (30), 526.3322 (5), 86.0968 (2), 60.0814 (2), 485.2559 (1)	−0.1	LysoPC (20:4 (8Z,11Z, 14Z, 17Z)) ^A, C, D^
16	23.8	0.13, 0.30	520.3392	[M + H] ^+^	104.1078 (34), 184.075 (33), 258.1124 (1), 337.2781 (1), 443.2566 (1)	1.1	LysoPC (18:2(9Z,12Z)/0:0) ^A, C, D^
17	25.5	0.24, 0.30	496.3398	[M + H] ^+^	184.0752 (100), 104.1076 (1)	−0.1	LysoPC (16:0/0:0) ^A, C, D^
18	26.6	0.13, 0.30	522.3556	[M + H] ^+^	104.1077 (33), 184.075 (30)	0.7	LysoPC (18:1(9Z)) ^A, D^
**II**	**Metabolites in urine**						
19	3.1	0.18, 0.10	114.0675	[M + H] ^+^	59.0505 (48), 86.0733 (10), 72.0458 (1)	−12.5	Creatinine ^A, D, F^
20	3.4	0.12, 0.20	144.1027	[M + H] ^+^	58.0664 (100), 84.0827 (26), 72.0826 (14)	−5.5	Proline betaine ^A, D^
21	7.5	0.06, 0.20	166.0870	[M + H] ^+^	120.0839 (100), 103.0566 (5), 131.0521 (4), 77.0406 (1)	−4.5	Phenylalanine ^A, E, F^
22	8.8	0.12, 0.30	318.2025	[M + H] ^+^	157.0898 (31), 85.0302 (13), 241.1129 (13) 60.0820 (9), 111.0832 (2), 274.1261 (1)	−0.5	Valine-leucine-Serine ^B^
23	9.1	0.06, 0.20	256.1915	[M + H_2_O] ^+^	85.0304 (86), 197.0503 (22), 57.0343 (5), 144.106 (4), 60.0821 (3)	−3.0	Heptanoylcarnitine ^A^
24	9.5	0.05, 0.19	232.0283	[M + H] ^+^	85.0306 (100), 173.0852 (25), 60.0821 (12)	−3.8	Benzeneacetamide-4-O-sulphate ^A^
25	13.6	0.08, 0.20	196.0611	[M + H] ^+^	121.032 (100), 65.0401 (2), 93.0757 (1)	−3.4	3-hydroxyhippuric acid ^A, D, E^
26	13.6	0.05, 0.20	432.2406	-	253.1501 (6), 85.0303 (2), 315.1423 (2), 297.1328 (1), 60.0813 (1), 144.1048 (1)	-	UI
27	13.9	0.11, 0.20	300.2178	-	85.0302 (96), 251.1331 (13), 121.1035 (13)	-	UI
28	14.7	0.05, 0.20	325.2272	-	86.098 (77), 91.0565 (51), 233.1712 (51), 85.0309 (9)	-	UI
**III**	**Metabolites in feces**						
29	2.8	0.08, 0.50	147.1143	[M + H] ^+^	84.0464 (100), 102.0572 (33), 130.0529 (33), 56.0507 (6), 85.0493 (5)	−4.0	Lysine ^A, F^
30	3.7	0.12, 0.30	130.0875	[M + H] ^+^	84.083 (100), 59.0741 (1)	−9.6	Cycloleucine ^A, F^
31	3.7	0.12, 0.30	160.1347	[M + H] ^+^	101.062 (26), 60.0822 (2), 55.0553 (11), 83.0509 (7)	−9.3	2-aminooctanoic acid ^A^
32	4.0	0.09, 0.40	118.0878	[M + H] ^+^	72.0824 (100), 59.0504 (29), 55.0553 (18)	−13.1	5-amino-pentanoic acid ^A^
33	4.1	0.08, 0.50	150.0596	[M + H] ^+^	56.0507 (100), 104.0551 (81), 133.035 (63), 61.012 (34), 87.0281 (8)	−8.5	Methionine ^A, F^
34	4.2	0.06, 0.40	182.0833	[M + H] ^+^	136.0756 (100), 165.0544 (59), 123.0441 (34), 147.0439 (15), 91.0541 (10)	−9.0	Tyrosine ^A, E, F^
35	4.7	0.09, 0.40	132.1032	[M + H] ^+^	86.0959 (100), 69.0693 (9), 59.0483 (1)	3.8	Leucine/isoleucine ^A, E, F^
36	6.2	0.06, 0.40	182.0828	[M + H] ^+^	136.0788 (100), 165.0585 (55), 123.0469 (34), 119.0518 (19), 147.0473 (15), 91.0562 (11)	−9.0	Tyrosine ^A, E, F^
37	5.6	0.05, 0.3	150.0596	[M + H] ^+^	104.0585 (100), 56.0526 (99), 133.0393 (65), 61.0139 (46), 87.0308 (8), 74.0274 (7)	−8.5	Racemethionine ^A, D^
38	6.9	0.32, 0.40	132.1002	[M + H] ^+^	86.0951 (100), 69.0686 (6)	12.8	Leucine/isoleucine ^A, E, F^
39	7.0	0.05, 0.40	264.1457	[M + H] ^+^	246.1352 (100), 200.1293 (62), 228.1245 (48), 131.0581 (16), 86.0968 (14)	-	UI
40	8.0	0.19, 0.40	166.0880	[M + H] ^+^	120.084 (100), 103.0567 (5), 131.0522 (3), 149.0633 (1)	−10.5	Phenylalanine ^A, E, F^
41	8.5	0.06, 0.30	229.1561	[M + H] ^+^	86.0977 (100), 116.0725 (97), 70.0662 (11)	−6.2	Leucyl-proline ^A, D^
42	9.2	0.10, 0.40	205.0988	[M + H] ^+^	188.0755 (100), 146.0635 (42), 118.0679 (7)	−8.0	L-tryptophan ^A, E, F^
43	12.8	0.11, 0.40	588.4181	-	566.4354 (100)	-	UI
44	15.5	0.05, 0.30	398.3456	-	98.0989 (1)	-	UI
45	19.1	0.06, 0.30	348.0668	-	111.0582 (100), 137.0380 (46), 180.0815 (28)	-	UI
46	20.4	0.05, 0.60	455.3547	[M + H] ^+^	369.2884 (16), 109.1039 (9), 437.3512 (9), 163. 0784 (3), 135.1195 (2)	−6.0	Jujubogenin/ jujubogen isomer [15]
47	23.0	0.06, 0.30	373.2754	[M + H] ^+^	355.2726 (100), 337.2612 (6), 245.1600 (5), 107.0880 (2)	−4.5	Cervonoyl ethanolamide ^A^
48	27.7	0.07, 0.60	455.3542	[M + H] ^+^	369.2884 (16), 437.3526 (10), 109.1036 (8), 123.1199 (8)	−4.9	Jujubogenin/ jujubogen isomer [15]
49	28.2	0.10, 0.60	473.3656	[M + H] ^+^	455.3624 (10), 437.3529 (7), 109.1041 (4), 123.1198 (3), 369.2888 (3)	−6.5	Psuedojujubogenin [15]

* The database and literature used for identification of selected features, where A = HMDB, B = METLIN, C = LIPID MAP, D = MetFrag, E = ChemSpider, F = Mass Bank; * UI- Unidentified.

## Data Availability

The data presented in this study are available on request from authors due to ethical issue.

## Supplementary Materials

The following are available online. Figure S1: Base peak chromatograms (BPC) from positive mode LC-ESI-QTOF-MS of (**a**) Brahmi essence and (**b**) placebo, where; BaA3: bacoside A3, Ba II: bacopaside II, BaX: ba-copaside X, BaC: bacopasaponin C and Ba I: bacopaside I, Figure S2: Scheme of sampling time points of biological samples throughout the study, Table S1: Some tentatively identified compounds in Brahmi essence and placebo analyzed by positive mode LC-ESI-QTOF-MS, Table S2: The percent change of response time for memory speed of participants from placebo run-in (2nd visit) to after 12 weeks (3rd visit) in treatment group and the placebo group, Table S3: *p*-value of metabolic pathway from enrichment analysis by using Metaboanalyst (a) plasma, (b) urine, (c) feces samples.

## Sample Availability

The samples presented in the paper are available on request from the authors.
