# Supplementary material for: Characterization of Metabolites in Plasma, Urine and Feces of Healthy Participants after Taking Brahmi Essence for Twelve Weeks Using LC-ESI-QTOF-MS Metabolomic Approach
Source: Molecules. 2021 May 15;26(10):2944. doi: 10.3390/molecules26102944 (PMC8156768; doi:10.3390/molecules26102944)
Supplement: Supplementary file 1 [file molecules-26-02944-s001.zip › molecules-1191054-supplementary.pdf]

Supplementary data

Characterization of Metabolites in Plasma, Urine and Feces of Healthy Participants After Taking Brahmi Essence for Twelve Weeks Using LC-ESI-QTOF-MS metabolomic approach

Genet Minale [1], Tongchai Saesong [1], Prapapan Temkitthawon [1], Neti Waranuch [2], Nitra Nuengchamnong [3], Krongkarn Chootip [4], Natakorn Kamkaew [4,5], Teeraporn Kongbangkerd [6], Jinutda Engsuwan [7] and Kornkanok Ingkaninan [1, *]

[1] Centre of Excellence in Cannabis Research, Department of Pharmaceutical Chemistry and Pharmacognosy, Faculty of Pharmaceutical Sciences and Center of Excellence for Innovation in Chemistry, Naresuan University, Phitsanulok 65000, Thailand; genetminaleye59@nu.ac.th (G.M.); tongchais56@nu.ac.th (T.S.); prapapant@nu.ac.th (P.T.)

[2] Cosmetics and Natural Products Research Center, Department of Pharmaceutical Technology, Faculty of Pharmaceutical Sciences, and Center of Excellence for Innovation in Chemistry, Naresuan University, Phitsanulok 65000, Thailand; netiw@nu.ac.th

[3] Science Laboratory Centre, Faculty of Science, Naresuan University, Phitsanulok 65000, Thailand; nitran@nu.ac.th

[4] Department of Physiology, Faculty of Medical Sciences, Naresuan University, Phitsanulok 65000, Thailand; krongkarnc@nu.ac.th (K.C.); natakorn.ka@up.ac.th (N.K.)

[5] Unit of Excellence in Clinical Research, Division of Physiology, School of Medical Sciences, University of Phayao, Phayao 56000, Thailand

[6] Department of Agro-Industry, Faculty of Agriculture Natural Resources and Environment, Naresuan University, Phitsanulok 65000, Thailand; teerapornk@nu.ac.th

[7] Department of Cosmetic Sciences, School of Pharmaceutical Sciences, University of Phayao 56000, Thailand; jinutda.en@up.ac.th

* Correspondence: k_ingkaninan@yahoo.com or kornkanoki@nu.ac.th; Tel.: +66-81-481-7350

Table of content

Figure S1. Base peak chromatograms (BPC) from positive mode LC-ESI-QTOF-MS of (a) Brahmi essence and (b) placebo where; BaA_3: bacoside A_3, Ba II: bacopaside II, BaX: bacopaside X, BaC: bacopasaponin C and Ba I: bacopaside I

Figure S2. Scheme of sampling time points of biological samples throughout the study.

Figure S3: The PCA score plots of A) plasma, B) urine and C) feces samples

Figure S4: The Base peak chromatogram of methanol (blank sample)

Table S1. Some tentatively identified compounds in Brahmi essence and placebo analyzed by positive mode LC-ESI-QTOF-MS.

Table S2. The percent change of response time for memory speed of participants from placebo run-in (2nd visit) to after 12-weeks (3rd visit) in treatment group and the placebo group.

Table S3. p-value of metabolic pathway from enrichment analysis by using Metaboanalyst (a) plasma, (b) Urine, (c) feces Samples.

.

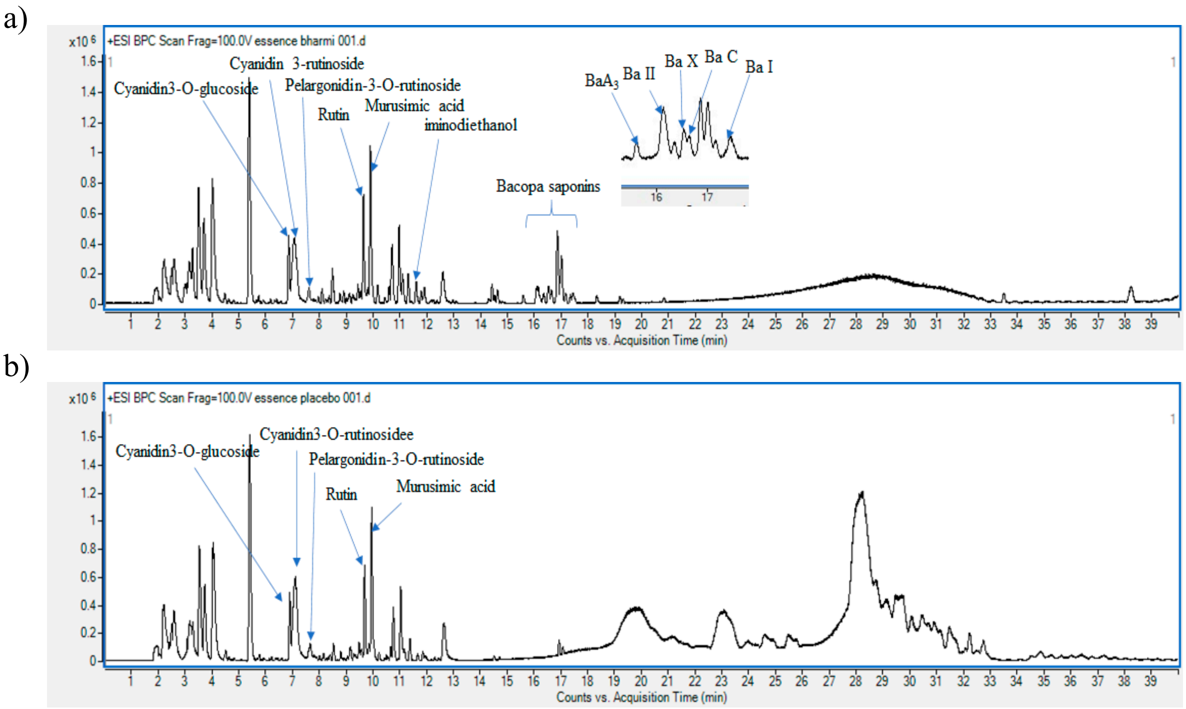

Figure S1 Base peak chromatograms (BPC) from positive mode LC-ESI-QTOF-MS of (a) Brahmi essence and (b) placebo where; BaA_3: bacoside A_3, Ba II: bacopaside II, BaX: bacopaside X, BaC: bacopasaponin C and Ba I: bacopaside I.

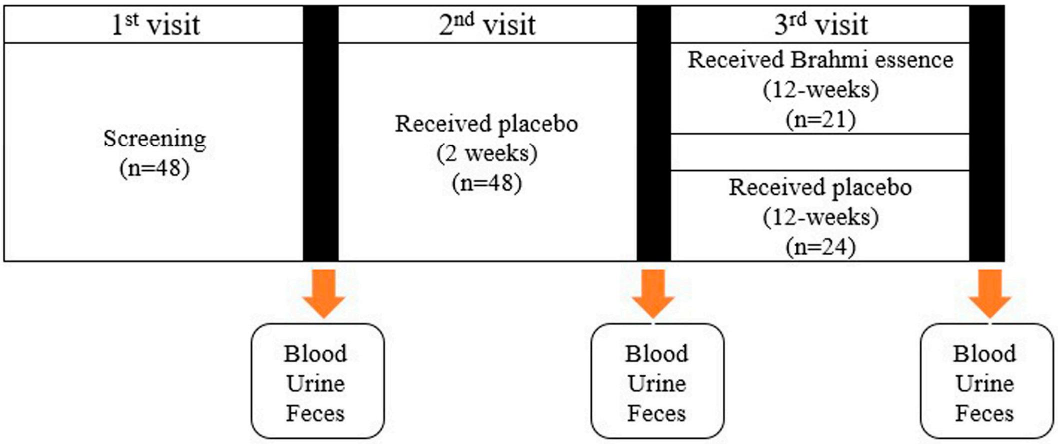

Figure S2: Scheme of sampling time points of biological samples throughout the study.

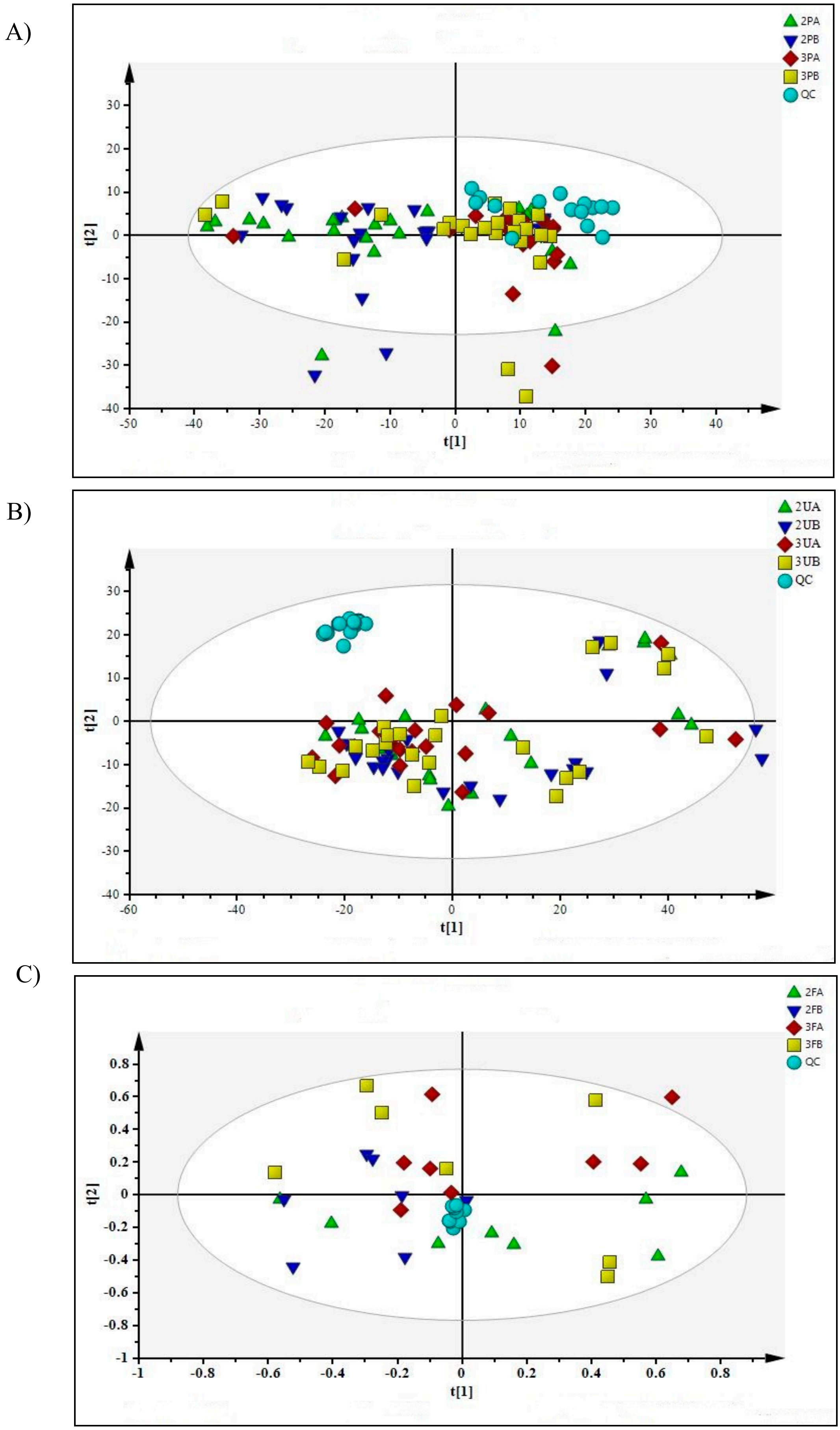

Figure S3: The PCA score plots of A) plasma, B) urine and C) feces samples. The data used for PCA were normalized by total peak intensities of each samples.

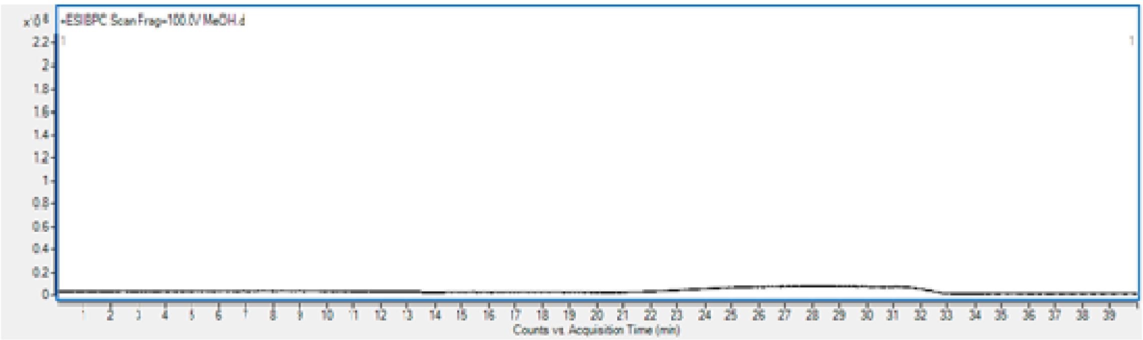

Figure S4: The Base peak chromatogram of methanol (blank sample)

Table S1. Some tentatively identified compounds in Brahmi essence and placebo analyzed by positive mode LC-ESI-QTOF-MS.

Rt (min)	Detected m/z	Assigned ion	Tentatively identified compounds	Samples
7.0	449.1093	[M+H] +	Cyanidin 3-glucoside	Brahmi essence, placebo
7.1	595.1683	[M] +	Cyanidin 3-rutinoside	Brahmi essence, placebo
7.6	579.1693	[M] +	Pelargonidin3-O-rutinoside	Brahmi essence, placebo
9.7	611.1632	[M+H] +	Rutin	Brahmi essence, placebo
9.9	492.3182	[M+H] +	Murusimic acid	Brahmi essence, placebo
11.6	218.2126	[M+H] +	iminodiethanol	Brahmi essence
15.6	951.4984	[M+H] +	Bacoside A_3	Brahmi essence
16.1	951.4954	[M+H] +	Bacopaside II	Brahmi essence
16.5	921.4855	[M+H] +	Bacopaside X	Brahmi essence
16.6	921.4850	[M+H] +	Bacopasaponin C	Brahmi essence
17.4	979.4601	[M+H] +	Bacopaside I	Brahmi essence

Table S2. The percent change of response time for memory speed of participants from placebo run-in (2nd visit) to after 12-weeks (3rd visit) in treatment group and the placebo group.

**Group A**		**Group B**	
**Subjects**	**% change of response time**	**Subjects**	**% change of response time**
1	6.37	1	20.36
2	-24.87	2	-4.30
3	-31.91	3	13.04
4	8.19	4	-4.27
5	1.36	5	-0.67
6	-2.71	6	-4.02
7	0.02	7	-9.87
8	-3.92	8	-16.86
9	4.27	9	6.97
10	-19.82	10	7.32
11	-22.22	11	8.03
12	4.24	12	-8.18
13	-4.32	13	7.93
14	-4.71	14	5.88
15	-30.72	15	-24.42
16	-3.31	16	-7.31
17	5.61	17	-9.37
18	-24.28	18	-13.28
19	-23.76	19	-0.35
20	0.50	20	-13.34
21	0.25	21	4.12
		22	17.46
		23	-12.03
		24	-7.70

Table S3. p-value of metabolic pathway from enrichment analysis by using Metaboanalyst (a) plasma, (b) Urine, (c) feces samples

(a)

Metabolic pathway	p value
Aminoacyl-tRNA biosynthesis	5.99E-05
Phenylalanine, tyrosine, and tryptophan biosynthesis	0.000538
Valine, leucine, and isoleucine biosynthesis	0.00245
Phenylalanine metabolism	0.0039
Valine, leucine, and isoleucine degradation	0.057
Ubiquinone and other terpenoid-quinone biosynthesis	0.0855
Pantothenate and CoA biosynthesis	0.172
Fatty acid degradation	0.324
Tryptophan metabolism	0.337
Tyrosine metabolism	0.344

(b)

Metabolic pathway	p value
Phenylalanine, tyrosine, and tryptophan biosynthesis	0.0131
Phenylalanine metabolism	0.0325
Aminoacyl-tRNA biosynthesis	0.148

(c)

Metabolic pathway	P value
Aminoacyl-tRNA biosynthesis	1.40E-08
Phenylalanine, tyrosine, and tryptophan biosynthesis	0.000339
Valine, leucine, and isoleucine biosynthesis	0.00156
Phenylalanine metabolism	0.00248
Valine, leucine, and isoleucine degradation	0.0376
Ubiquinone and other terpenoid-quinone biosynthesis	0.0689
Biotin metabolism	0.0763
Lysine degradation	0.181
Cysteine and methionine metabolism	0.232
Tryptophan metabolism	0.28
Tyrosine metabolism	0.286
Fatty acid biosynthesis	0.315
